# An update on AI in Academic Publishing

**DOI:** 10.1051/ject/2026023

**Published:** 2026-06-19

**Authors:** Raymond K. Wong

Artificial intelligence (AI) is being discussed everywhere and is increasingly influencing many aspects of our lives, including technology, business and investment, education, and, indeed, scientific publishing. The pace of change can feel overwhelming, yet it is essential that we remain engaged. Despite its many potential benefits, AI also presents risks that could negatively affect our missions and professional endeavors. We must understand how to use AI safely and appropriately while guarding against its potential adverse effects.

I wrote an editorial several years ago when the AI phenomenon was just beginning to emerge [1]. Three years later, an update is timely, as additionally nuanced guidance has become available from academic publishing advocacy organizations, including the Committee on Publication Ethics (COPE), of which the Journal of Extra-Corporeal Technology (JECT) is a member.

The challenges posed by AI in publishing begin with the potential erosion of academic integrity and, in clinical journals, the possible impact on patient safety. AI can also contribute to the publishing equivalent of spam email – large volumes of manuscript submissions that may overwhelm editorial workflows and make it more difficult to identify inappropriate or erroneous content. In addition, AI may be inappropriately used by peer reviewers and editors, further compromising the integrity of the publication process.

As AI technology continues to advance, detecting its use throughout the publishing workflow will become increasingly difficult. However, should all use of AI be prohibited, or should all use be disclosed? This is where guidance from professional organizations can help. Based on recommendations summarized by COPE, several key principles include:

1. The use of basic author-support tools generally does not require disclosure; however, more substantive use of AI should be disclosed.
2. Disclosure is intended to promote transparency and is not meant to stigmatize or shame authors.
3. The use of AI by peer reviewers or editors on public platforms constitutes a breach of confidentiality. The use of secure, proprietary systems that maintain confidentiality may be acceptable but should be disclosed.
4. AI should never be used to make final decisions regarding manuscript disposition.

There is much more very helpful content publicly available on COPE’s website, for those who are interested [2].

Currently, the only AI-related technology deployed by JECT is iThenticate (Turnitin LLC, Oakland, CA, USA), which is used to conduct plagerism/similarity checks. However, this technology is not designed to detect the use of generative AI by authors. For the foreseeable future, we will continue to rely on our peer reviewers to identify inappropriate or erroneous uses of AI in manuscripts, including hallucinated content, inaccurate references, and other related concerns. For my part, I will continue to share relevant recommendations, guidance documents, and best practices with our peer reviewers and editors as they become available. In the meantime, I invite feedback and comments regarding any aspect of AI use within JECT. Whether you are a reader, author, peer reviewer, editor, or member of our editorial board, your perspectives and insights are welcome.

While current guidance suggests that routine use of basic author-support AI tools need not be disclosed, the following declaration of generative AI use, prepared with assistance from ChatGPT, is presented as an example of the transparency statement that authors should consider including when generative AI has played a more substantial role in the development, drafting, or revision of a manuscript.

## Declaration of generative AI use

Generative artificial intelligence (AI) tools, specifically ChatGPT (OpenAI, San Francisco, CA, USA), were used during the preparation of this editorial to assist with language refinement, grammar, style, and readability. All content was reviewed and edited by the author, who accepts full responsibility for the accuracy, integrity, and final version of the text.
